# Legal and systemic implications of the 2023 Polish act on certain medical professions–regulation, workforce requirements, healthcare system impact

**DOI:** 10.3389/fpubh.2025.1679450

**Published:** 2025-11-11

**Authors:** Jolanta Agnieszka Pacian, Marlena Jolanta Piekut

**Affiliations:** 1 Department of Medical and Pharmaceutical Law, and Psychosocial Aspects of Medicine, Medical University of Lublin, Lublin, Poland; 2 College of Economics and Social Sciences, Warsaw University of Technology, Warsaw, Poland

**Keywords:** healthcare regulation, medical professions, professional development, health workforce policy, health law, Poland, patient safety, health systems governance

## Abstract

**Background:**

In August 2023, Poland enacted the Act on Certain Medical Professions, introducing binding legal requirements for 15 previously unregulated health professions. The Act mandates continuous professional development (CPD) and registration in the Central Register of Persons Authorized to Practice a Medical Profession. Its primary aim is to enhance legal accountability, standardize qualifications, and improve healthcare quality and safety.

**Objective:**

The aim of this article is to provide a comprehensive assessment of the 2023 Act on Certain Medical Professions, including an analysis of the effectiveness of its legal provisions in strengthening professional accountability and patient safety, as well as an evaluation of its potential economic implications for the healthcare system.

**Methods:**

Employing a normative-descriptive approach, the study conducts a legal and policy analysis and includes a comparative overview of similar regulatory models in selected EU countries.

**Results:**

The Act formalizes these professions and introduces significant legal and organizational duties. While it may result in short-term costs—such as those related to ICT systems, registration, and CPD—it offers long-term benefits through improved care quality, reduced medical errors, and enhanced patient trust.

**Conclusion:**

The Act marks a major reform in Polish healthcare regulation. Despite higher compliance demands, it strengthens professional standards and system efficiency. Future empirical studies should examine its long-term impact.

## Highlights

- *What is already known about the topic*? Across the EU, medical professions are governed by national legal frameworks regulating access, qualifications, and professional obligations. Countries such as Latvia, Lithuania, and the Czech Republic have implemented laws covering certification, registration, and continuing professional development (CPD), typically aligned with Directive 2005/36/EC on the recognition of professional qualifications.
- This article provides the first comprehensive legal and economic analysis of the Polish Act of 17 August 2023 on Certain Medical Professions. The Act introduces two key statutory obligations—mandatory registration in a central public register and compulsory CPD—for fifteen previously under-regulated health professions.
- *What does this study add to the literature*? The article combines doctrinal legal analysis with health policy and economic perspectives, offering an interdisciplinary assessment of the new law’s implications. It contributes to the literature by highlighting how formal legal regulation supports professional accountability, enhances transparency, and enables quality control in healthcare delivery.
- *What are the policy implications*? While implementation may entail short-term costs related to compliance, administration, and training, the long-term benefits include improved systemic efficiency, reduced medical errors, and stronger public trust. The new regulatory framework also creates conditions for more equitable healthcare financing and better labor market integration. The study has broader relevance for policymakers in high-income countries regulating emerging health professions.

## 1 Background

The Act of 17 August 2023 on Certain Medical Professions (ACMP) (1), in force since 26 March 2024, introduced key regulatory changes to the Polish healthcare system. It defines fundamental standards for the practice of 15 specified medical professions, establishing both rights and obligations for practitioners and significantly broadening their legal liability.

The primary objective of this article is to provide a comprehensive assessment of the 2023 Polish Act on Certain Medical Professions, combining two complementary perspectives:

(1) a legal analysis of the effectiveness of its regulatory instruments in strengthening professional accountability, improving the quality of care, and ensuring patient safety, and
(2) an economic evaluation of the anticipated impacts on the healthcare system, labor market, and public expenditure.

The central research question is therefore: *How does the Act balance legal-regulatory effectiveness with its economic consequences for the Polish healthcare system?* Specific research issues include: standards of professional conduct necessary to avoid liability; obligations to inform patients of their rights; conditions for refusing service provision; supervision requirements for returning practitioners; and the legal nature of the Central Register of Persons Authorized to Practice a Medical Profession.

A major challenge in analyzing the Act lies in its linguistic imprecision and the need to adopt certain interpretative assumptions *a priori*, which must nonetheless be treated as legally binding.

Economically, a crucial issue is the effect of statutory rights and duties on healthcare system performance. The Act regulates 15 professions: dental assistant, electroradiologist, dental hygienist, addiction therapy instructor, medical caregiver, optometrist, orthoptist, podiatrist, prophylaxis specialist, hearing aid technician, pharmaceutical technician, massage therapist, orthopedic technician, medical sterilization technician, and occupational therapist.

A notable gap concerns dietitians, who are covered only when providing services financed from public funds under Article 5(35) of the Act on Healthcare Services Financed from Public Funds (2). As a result, identical professional activities performed in private practice fall outside the Act’s scope, creating regulatory asymmetry. This inconsistency may cause unequal market conditions and complicate public spending oversight. It also means that professionals doing the same work may face different legal obligations simply because of how their services are financed.

Comparative analysis shows that similar regulatory models exist in EU member states. In Latvia, medical professions are governed by the *Law on Medical Treatment* (3); Lithuania regulates them under the *Law on the Recognition of Regulated Professional Qualifications* (4); while the Czech Republic employs a mixed model via Act No. 48/1997 Coll. and supplementary acts (5–7). Despite national differences, these frameworks are underpinned by EU Directive 2005/36/EC on the recognition of professional qualifications (8). Further comparative evidence can be drawn from other OECD countries. For instance, in the UK, the introduction of mandatory medical revalidation has formalized appraisal systems and significantly enhanced the monitoring of professional practice, though comprehensive outcome metrics are still being evaluated (GMC, UMbRELLA report) (9, 10). In Sweden, recent labor market reforms include substantial publicly funded CPD and retraining—estimated at around SEK 11 billion annually—aimed at strengthening workforce skills and adaptability (11). Additional OECD evidence supports this trend. For example, Denmark’s 2018 Health Personnel Act introduced mandatory digital registration and continuous education programs, initially costing around EUR 25 million but resulting in measurable reductions in administrative errors and workforce attrition (12, 13). Similarly, in the Netherlands, national re-certification for allied health professions has led to long-term savings through improved quality control and integration with electronic health systems (14).

Thus, the Polish Act of 2023 aligns with broader European trends toward standardizing and formalizing medical professions within a unified regulatory structure.

This study uses a qualitative, normative-descriptive approach based on legal and policy analysis of the 2023 Act. It combines doctrinal methods, focused on statutory provisions, legal doctrine, and case law, with a policy-oriented perspective examining the Act’s economic and organizational implications for the healthcare system. The normative-descriptive approach was applied in three steps. First, sources were selected based on their legal relevance (statutory acts, parliamentary records, case law), credibility (peer-reviewed commentaries, official reports), and timeliness (2010–2024). Second, where possible, comparative references to other countries were incorporated. Third, the findings were synthesized thematically, combining doctrinal interpretation with an assessment of systemic and economic implications.

The data sources used in this study are secondary and publicly available. These include national legislation, European Union directives, parliamentary documentation, and legal commentaries. The research is retrospective and cross-sectional in nature. Comparative elements are also included.

From an economic perspective, the implementation of the Act involves both short-term expenditures and potential long-term savings. The preliminary cost estimates presented in this article were derived from publicly available data, including parliamentary budget documents, official statements of the Ministry of Health, and comparative OECD and Eurostat indicators (15–21). These estimates should therefore be interpreted as indicative rather than definitive.

For analytical purposes, costs were categorized into three groups: (1) infrastructure and ICT expenditures (including system development, cybersecurity, and interoperability); (2) recurrent training and CPD costs; and (3) administrative and staffing expenses related to registration and monitoring. The projections assume initial investment costs of approximately PLN 40–60 million for ICT infrastructure, with annual maintenance of PLN 4–6 million, and CPD-related costs of PLN 50–150 million per year (16–18).

When assessed over a medium-term horizon of five to 10 years, these costs may be offset by efficiency gains arising from reduced hospital readmissions, improved professional competence, and lower rates of medical error. Simulation models based on OECD and Eurostat data suggest that a 2.5–5% reduction in avoidable readmissions could generate annual savings of PLN 70–200 million in public healthcare expenditure (15, 19–21). Consequently, the reform may prove fiscally neutral—or even cost-saving—within a decade of full implementation.

The economic implications also extend to the private healthcare sector. While private providers are expected to bear part of the compliance and training costs, they may benefit from improved service quality, reduced malpractice risks, and greater patient trust. The combined effect of these factors is likely to enhance overall market transparency and stimulate fair competition between public and private healthcare entities.

## 2 Formal and legal requirements for practicing a medical profession under the ACMP

Article 2(1) of the ACMP (22) sets out the formal and legal prerequisites for practicing one of the 15 regulated medical professions. These include: full legal capacity to act; no final conviction for intentional offenses prosecuted by public indictment or for fiscal crimes; full public rights; and proficiency in Polish, confirmed by a formal statement or acquired through Polish-language education or training. Additionally, practitioners must possess relevant education or qualifications specified in the Annex to the Act or recognized through EU or international agreements, and must be registered in the Central Register of Persons Authorized to Practice a Medical Profession.

The requirement of full legal capacity, common to other medical professions such as physicians and nurses, also applies to roles like health prevention specialists and occupational therapists. As Włodarczyk notes, legal regulation requires identifying who has the passive and active legal capacity to enter and perform in legal relationships (23). Loss of such capacity—e.g., through incapacitation caused by mental illness or addiction—results in automatic disqualification from professional practice.

The Act also requires a clean criminal record and enjoyment of public rights, aligning with standards for paramedics under Article 2(6) of the 2022 Act on the Profession of the Paramedic (24). However, paramedics must additionally demonstrate that their prior conduct guarantees ethical performance—stricter than the requirements for physicians and nurses, who must prove relevant education, capacity to act, ethical standing, and health condition (25, 26). This raises concerns about the proportionality of imposing such rigorous criteria on non-autonomous roles like occupational therapists, while ethical conduct is not listed among formal prerequisites for any of the 15 professions under ACMP.

Although ethical breaches entail professional liability, the absence of ethical standards at the entry stage is problematic. It remains unclear whether this is a legislative omission or an intentional choice. De lege ferenda, the ACMP should be amended to ensure ethical conduct is a prerequisite for these professions, in line with patient safety considerations.

Economically, registration in the Central Register affects healthcare quality and the cost-efficiency of labor market access. While delays in registration may defer employment and reduce early-career income, the cost (100 PLN) and processing time (up to 3 months) are relatively low and unlikely to deter candidates. At the macroeconomic level, however, the creation and operation of the Central Register entails substantial costs. Development of the ICT platform and ensuring cybersecurity are estimated at 40–60 million PLN, with recurring annual expenses of 4–6 million PLN (17). These expenditures reflect the need for interoperability, digital security, and system upgrades.

## 3 Professional practice with due diligence, in accordance with current medical knowledge and skills

Each person practicing a medical profession is obligated to perform their duties with due diligence, in accordance with current medical knowledge and the skills necessary for their profession, while respecting patients’ rights and ensuring their safety (27, 28) (Art. 15 of the ACMP). To discuss this requirement further, it is essential to clarify the concept of *due diligence*. As R. Kędziora explains, this criterion is objective and abstract (29). In practice, it involves identifying a socially accepted model of optimal conduct under specific circumstances and comparing the practitioner’s actions to that standard (30). Liability may arise not only from a departure from this model but also from a failure to foresee the likely consequences of such behavior (31).

Economically, failure to meet the due diligence standard leads to increased system costs. Medical errors—often linked to a lack of diligence—generate expenses from complications, extended hospitalizations, and litigation (32). In the U. S., such errors cost the system an estimated USD 20 billion annually (33). In Poland, compensation claims range from PLN 10,000 to over PLN 1 million (EUR 2,361 – EUR 236,183) (34).

To mitigate these risks, profession-specific conduct models are developed—such as the “good doctor” or “good nurse.” Analogous models should guide other medical professionals like prevention specialists, dietitians, occupational therapists. These models help assess both competencies and obligations, especially regarding the foreseeability of consequences resulting from negligence. Establishing such standards enables uniformity in fulfilling the requirement to act with due diligence and apply current medical knowledge, thereby ensuring patient safety and quality of care (35).

The introduction of *evidence-based practice* (EBP) reinforces these standards. EBP not only improves treatment outcomes but also enhances cost-efficiency by limiting unnecessary procedures and hospitalizations (36). Thus, embedding EBP into the professional responsibilities of healthcare practitioners reflects both legal and economic imperatives.

A separate but related obligation imposed by Article 15 of the Act is the provision of services aligned with current medical knowledge. As A. Fiutak notes, this concept goes beyond codified scientific knowledge and includes *praxis*—practical experience that co-shapes the epistemic base of medical science (37). The requirement implies a duty to engage in methodologically aware, falsifiable, and verifiable practice (38). While “current medical knowledge” is globally objective, its application must be context-sensitive, factoring in both the specific medical case and the practitioner’s qualifications. The patient’s right to treatment in line with such knowledge must be understood in this individualized context.

From an economic angle, adherence to current medical knowledge enhances cost-effectiveness in healthcare delivery (15, 39). For instance, type 2 diabetes patients treated according to American Diabetes Association (ADA) guidelines incur lower healthcare costs than those treated otherwise (40). Similarly, EBP in rehabilitation has been shown to improve care quality while addressing the financial pressures on the system (41). Avoiding ineffective methods reduces rehospitalization, a major driver of medical costs. Further, as Dizon et al. argue, integrating EBP and quality standards into medical education improves health outcomes and supports cost monitoring per patient, thereby assisting in the prevention of avoidable readmissions (42).

These legal and economic rationales are not unique to the 15 professions covered by the Act. They mirror provisions found in other statutes governing the practice of physicians. Article 4 of the Act on the Medical Profession outlines obligations to act in line with current medical knowledge, adhere to professional ethics, and exercise due diligence. However, it notably omits an explicit reference to the obligation to act within legal boundaries—despite Article 53 of the same Act imposing professional responsibility for legal violations. This omission seems intentional, as physicians, like all citizens, are presumed to comply with applicable law without such a reminder. Yet, explicitly citing legal violations as grounds for disciplinary action emphasizes their relevance within the framework of professional liability (43).

These same principles apply *per analogiam* to the 15 medical professions specified in the ACMP. Although these roles may not carry the same level of institutional autonomy as physicians or nurses, the obligations toward diligence, evidence-based practice, and legal compliance are equally binding. Uniform expectations in legal responsibility and professional standards strengthen public trust and optimize the quality and efficiency of healthcare delivery.

## 4 Obligation to inform the patient of their rights and to comply with them

Pursuant to Article 16 of the ACMP, a person practicing a medical profession is obliged to comply with patients’ rights (44) and to inform patients of their rights in accordance with the Act of 6 November 2008 on Patients’ Rights and the Patient Ombudsman (45). Every medical professional is thus under a dual obligation: to observe (46) and to inform patients of their rights (47). Fulfilling the duty of providing information is a prerequisite for the legality of any action taken by a healthcare professional (48, 49). Therefore, it is essential to provide the patient with information about their health condition and therapeutic plan, enabling them to participate consciously and actively in the treatment and care process (50). This duty includes communicating with the patient (51) in a manner that is clear, comprehensible (52, 53), and adapted to their intellectual capabilities, using words, terms, and names that are simple and understandable. One must bear in mind that a medical professional only fulfills their duty when the patient *understands* (54) the content of the message—not just *hears* it, as J. Jabłońska rightly emphasizes (55, 56). If the healthcare professional notices that the patient has not fully understood the information, they should repeat the explanation, address doubts, and clarify unclear points (57). It is essential that the patient clearly understands what the healthcare professional – for example, a dietitian or occupational therapist – is communicating, as well as the nature of the proposed service. Such understanding enables the patient to make an informed decision by weighing benefits, drawbacks, and possible risks.

From an economic standpoint, the duty to inform and respect patients’ rights affects not only the quality of care but also reduces costs stemming from litigation, compensation claims, or communication errors. Research shows that improving communication between healthcare staff and patients leads to fewer legal disputes and, consequently, significant savings within the healthcare system (58–60).

However, the Act omits legal provisions regarding the duty of professional secrecy and exceptions to that duty (61). Although such provisions are contained in the Act on Patients’ Rights and the Patient Ombudsman, their omission from the present act is legally unjustified. This is because there is no obligation on the part of the patient to consult regulations found in other legal acts, which often leads to low levels of knowledge and awareness in this area.

The obligation to inform the patient (62) is directly connected with the issue of *consent* (63) to medical procedures (64). As K. Gibiński and J. J. Rybicka rightly note, “before the patient signs the consent form, a dialog between the doctor and patient (65) regarding the case must occur; a paternalistic monolog by the doctor is unacceptable.” (66). The Supreme Court has rightly stated that “a patient cannot be treated as an object of decisions made by others and cannot be deprived of the freedom to decide about their own health.” (67, 68). According to the Court, this does not mean that a doctor must inform the patient about all possible consequences of a procedure. The doctor is only required to provide the information necessary for the patient to give informed consent. This includes the type and purpose of the procedure, its positive and negative effects, and the likelihood of complications.

Informing the patient of *all* possible consequences could unnecessarily worsen their emotional state and lead to an unfounded refusal to undergo treatment. Therefore, doctors should not be required to inform patients about every potential complication, especially rare ones (69–76). The extent of the informational duty should be based on what a reasonable person in the patient’s situation objectively needs to know to make an informed and prudent decision (77). Hence, the content of the information should be tailored to the patient’s age, intellectual level, and mental state.

## 5 Need for and right to refuse to perform a healthcare service

If there are justified doubts regarding the execution of an assigned healthcare service, a medical professional has the right to request justification from the issuer of the order and also the right to refuse to perform the service. Any refusal must be documented in the medical records, and the ordering party must be informed (Art. 17 of the Act).

It should be emphasized, prima facie, that a medical professional has a statutory right to refuse to provide a healthcare service. This means that in justified cases, they may normatively refrain from performing the service (78). However, two additional obligations are imposed on the medical professional (79): first, to inform the ordering party; second, to meticulously record the case in the medical documentation.

Economically, the right to refuse may serve to rationalize healthcare costs, especially in cases where a service is medically or ethically unjustified. Avoiding low-value or high-risk procedures reduces unnecessary expenses, limits legal risk, and improves resource allocation (80). Moreover, enforcing this right may reduce burnout among healthcare personnel, which has measurable economic consequences, such as absenteeism, staff turnover, and decreased productivity (81).

Such a refusal may be justified by invoking the *conscience clause*. In such a case, the medical professional may refuse to perform the service. However, like physicians, they are obligated to provide assistance when it is urgently needed. As K. Piech rightly notes, this does not apply to situations where the patient could safely receive assistance at another time (82, 118). Therefore, if immediate care is not required, the service may be provided at another facility or by another professional. This solution is valid both medically and legally.

## 6 Interruption in practicing a medical profession

Anyone who has not practiced a medical profession for more than 5 years within the last 6 years, and intends to resume practice, is obliged to work under supervision for 6 months (Art. 19 of the Act). As D. Karkowska correctly points out in reference to nurses and midwives, “a serious flaw in current legal standards is that nurses cannot appeal decisions of regional nursing councils regarding the location, duration, or training program. These decisions are not made in the form of resolutions (a contrario Art. 40 of the Act on the Profession of Nurse and Midwife)” (83).

Moreover, the Supreme Court ruled on 30 January 2002 that “any refusal by a regional nursing council to assist in organizing retraining, and thus enabling the resumption of the nursing profession after a five-year break, may justify civil liability of council members under tort law.” (84). In its decision of 21 February 2007, the Supreme Court also held that retraining is not required if the nurse or midwife completed undergraduate or graduate studies in nursing or midwifery during the break (including both regular and supplementary programs) (85).

Although a regional council may refuse to assist in retraining, nurses and midwives who completed a degree during a break of more than 5 years may be exempted from this obligation. The Supreme Court’s legal interpretation in this matter is entirely justified and favorable to nurses and midwives. By analogy, this should also apply to the ACMP. Conversely, nurses and midwives who did not undertake education during the break must undergo retraining under the supervision of a specialist.

From an economic perspective, exempting those who pursued formal medical education during a break from retraining prevents unnecessary costs and helps reduce staffing shortages caused by delays in returning to practice (86, 87). Applying *a maiori ad minus* reasoning, if completing a higher education degree in nursing or midwifery exempts one from retraining, then completing at least one form of postgraduate medical education—such as a specialization, supplementary, specialist, or qualification course—should also justify exemption.

De lege ferenda, a similar legislative proposal should be made regarding the 15 medical professions defined in the ACMP, such that completing medical studies or postgraduate education during a career break would constitute a *sine qua non* condition for exemption from mandatory retraining. This proposal aligns with the principle of effective human resource management in healthcare (88, 89).

## 7 The legal nature of the CRPAPMP

A major reform introduced by the ACMP is the creation of the Central Register of Persons Authorized to Practice a Medical Profession (CRPAPMP). This public register allows verification of qualifications for professionals in 15 regulated medical professions and enhances transparency for patients and healthcare providers. Unlike registers for doctors or nurses, the CRPAPMP is not maintained by a professional self-government, as no chambers have been established for these professions. Consequently, registration does not entail automatic membership in any self-governing body, which removes institutional constraints on professional activity.

The register serves both patients and healthcare institutions, ensuring that only qualified individuals provide services. It qualifies as a public register under Article 3(5) of the Act on the Computerization of Activities of Entities Performing Public Tasks and is maintained by the Minister of Health. Registration is conducted electronically and requires submission of an application, correction of any formal deficiencies within 21 days. Once verified, the applicant receives an individual registration number (Articles 3–12 of the Act) (90).

By 26 September 2024, all practitioners must be registered. Non-compliance beyond 25 March 2025 may result in a fine of up to PLN 5,000 or restriction of liberty, thus ensuring enforcement of the obligation.

## 8 The right and obligation of continuing professional development (CPD)

The Act also introduces a dual obligation and right to undertake continuing professional development (CPD) (Article 20). CPD includes both structured education and self-directed learning aimed at updating knowledge and skills. This requirement aligns with the principle that “medicine evolves rapidly” and maintaining current knowledge is crucial to ensure effective and safe patient care (91). From a financial perspective, CPD constitutes one of the largest recurrent costs introduced by the Act. Accredited training averages 1,000–3,000 PLN per professional per year (18). With approximately 50,000 practitioners covered, this translates into 50–150 million PLN annually. Costs are shared between healthcare institutions, employers, and in part may be co-financed by EU structural funds (16).

CPD obligations, derived also from Article 18 of the Act on the Professions of Physicians and Dentists, are legal duties as well as ethical imperatives. They aim to maintain high standards of professional conduct. The Act obliges public authorities and employers to support this process (92).

CPD encompasses two key categories: (1) advanced training courses whose curricula are developed by the Center of Postgraduate Medical Education, and (2) individual self-education.

## 9 Formal postgraduate education opportunities

Medical professionals may also participate in postgraduate education through specialist training (under the Act of 24 February 2017) or qualification courses (Article 22). The latter are intended to equip experienced professionals—those with at least 3 years in the field—with skills for specific tasks (Article 23).

Economically, CPD is an investment in human capital, consistent with the theories of T. W. Schultz and G. S. Becker (93). It increases the productivity and employability of healthcare professionals and improves the quality of services provided (16). A failure to update knowledge results in opportunity costs, greater risk of medical error, higher treatment costs, and legal exposure for employers (94).

Higher qualifications are also correlated with increased earnings and professional mobility (95). For healthcare institutions, CPD reduces staff turnover and recruitment costs (96, 97).

The term “professional development” under Article 61(1) of the Act on the Professions of Physicians and Dentists includes all forms of post-licensure education except postgraduate internships (98, 99). However, this exclusion is debated. Encyclopedically, professional development is the pursuit of excellence and better socio-economic positioning through skill enhancement (100). It is not necessarily tied to an employment relationship (101).

The Act’s current definition of CPD is narrow. De lege ferenda, the inclusion of postgraduate university studies as part of CPD should be considered.

The key macro- and microeconomic effects resulting from mandatory continuing professional development and registration in the CRPAPMP are summarized below (Table 1).

**Table 1:** Systemic and individual impacts of continuing professional development (CPD) and mandatory registration.

Dimension	Observed/Expected Effect	Supporting literature
Macroeconomic	Shorter hospitalizations Fewer misdiagnoses Reduction in unnecessary treatments More efficient public spending	(102–104)
Microeconomic	Improved labor market resilience Lower risk of professional exclusion	(105–107)
Systemic Adaptability	Enhanced responsiveness to demographic and epidemiological shifts	(108, 109)
Cost–Benefit Perspective	High short-term costs: ICT infrastructure (40–60 mln PLN initial, 4–6 mln PLN annual) CPD programs (50–150 mln PLN annual) Staffing/administration (10–20 mln PLN annual) Long-term efficiency and quality gains: Savings from reduced readmissions (70–200 mln PLN annually) Lower litigation costs Workforce productivity improvements	(16–19, 110)
Regulatory Function	Registration and CPD increase service quality Ensure professional competence	(111–113)
Consumer Confidence	Transparency improves trust Greater willingness to seek and pay for services	(114, 115)
Quality Assurance	Competence evaluation protects against substandard care Leads to better patient outcomes and satisfaction	(116, 117)

## 10 Conclusion

In conclusion, the formal and legal requirements for practicing one of the 15 medical professions regulated by the ACMP are generally standard, with two notable exceptions: the legal obligation of continuous professional development and mandatory registration in the CRPAPMP, classified as a public register. These provisions reflect the complexity and layered character of the Act and are justified under the current legal framework.

The first requirement—ongoing improvement of professional qualifications through postgraduate education and continuous development—is both a legal duty and a professional right. It ensures the maintenance of high standards in healthcare and serves the public interest by safeguarding the quality of medical services.

The second requirement introduces regulated access to these professions, emphasizing their social importance and simultaneously imposing stricter legal obligations on practitioners. This strengthens accountability and professional integrity.

From a legal and economic standpoint, the Act of 17 August 2023 carries important implications for the Polish healthcare system. It directly affects human resource planning, workforce management, and the financial dimensions of training and staff development. Regulatory formalization may also enhance public trust in medical services.

Introducing an obligation under the Polish Act on Certain Medical Professions to provide warnings through the IMI system would primarily benefit potential patients. This applies especially to those who might receive services from individuals performing these professions. The provisions on administrative cooperation contained in EU acts implemented through the IMI system include Regulation (EU) No 1024/2012 of the European Parliament and of the Council of 25 October 2012 on administrative cooperation through the Internal Market Information System and repealing Commission Decision 2008/49/EC (“the IMI Regulation”) (OJ L 316, p. 1). The legal basis also comprises Commission Decision 2009/739/EC of 2 October 2009 setting out the practical arrangements for the exchange of information by electronic means between Member States pursuant to Chapter VI of Directive 2006/123/EC of the European Parliament and of the Council on services in the internal market (OJ L 263, 7 October 2009, p. 32). Under this obligation, competent authorities in other EU Member States would provide information about a person practicing an allied health profession if a decision had been made to suspend or restrict their professional rights, or if a preventive measure had been issued by a court.

This information would concern individuals whose right to practice has been restricted in any way in another EU country or in the Swiss Confederation. Although for medical professions such as physicians or nurses this obligation already exists under current Polish regulations, the professional self-governing bodies of physicians, dentists, and nurses have the ability both to transmit and to receive information related to the procedure for recognizing professional qualifications.

This obligation should also apply to individuals practicing allied health professions who were trained abroad and subsequently apply for professional authorization in Poland. It should likewise apply to those seeking recognition in Poland of a specialization obtained abroad, as well as to Polish professionals intending to work in another country based on the recognition of qualifications obtained in Poland.

Inclusion of new professions in the regulatory framework entails costs related to maintaining the central register, ensuring digital infrastructure, funding mandatory training, and adjusting remuneration systems and career progression models in healthcare institutions. Preliminary estimates based on available Polish and international data suggest that the Act may have measurable fiscal implications. According to national hospital data, the rate of 30-day readmissions in Poland has been reported at approximately 12.5% (20) and in some settings even higher, reaching 19.2% (21). OECD and Eurostat statistics indicate that Poland records over 7 million hospital discharges annually, with an average cost per case of roughly 4,000–5,000 PLN (15, 17).

Assuming that improved regulation, registration, and continuing professional development contribute to a reduction in avoidable readmissions by just 5%, potential annual savings could reach 150–200 million PLN in public healthcare expenditure. Even more modest effects (a 2.5% reduction) would generate savings in the range of 70–100 million PLN.

These estimates were obtained using a simple cost-multiplication model based on publicly available OECD and Eurostat data. With approximately 7 million annual hospital discharges in Poland and an average treatment cost of around PLN 4,000–5,000 per case (17, 19), each 1% reduction in readmissions corresponds to roughly PLN 280–350 million in potential annual savings (7,000,000 × 0.01 × 4,000–5,000). Consequently, a 2.5–5% reduction would theoretically translate into total savings of PLN 0.7–1.6 billion per year.

However, because not all readmissions are fully preventable and a large portion of hospital expenditures are fixed rather than variable, the effective fiscal impact is expected to be substantially smaller. For this reason, the main text presents a conservative estimate of PLN 70–200 million in potential annual savings—representing only the realistically avoidable portion of readmission-related costs.

In macroeconomic terms, the potential savings of PLN 0.7–1.6 billion per year represent approximately 0.4–0.9% of the annual public healthcare budget in Poland. Although this share may seem modest at the national level, it equals the entire annual funding of major national health programs, such as child psychiatry or vaccination. At the institutional level, such savings correspond to the yearly budgets of two to three regional hospitals, making the fiscal implications substantial in operational terms. The magnitude of potential efficiency gains is therefore consistent with the 0.3–1% range observed in OECD countries following comparable regulatory and quality reforms (9–13, 15, 39).

These figures should be treated as illustrative simulations rather than precise forecasts; however, they highlight that the Act’s medium-term impact may be at least fiscally neutral and potentially cost-saving. Such projections align with international evidence showing that strengthening professional standards and introducing mandatory continuing education reduces adverse events and readmissions, thereby improving both quality of care and system efficiency (15, 39).

Comparative experience from OECD countries confirms this trend: in the UK, mandatory revalidation has enhanced professional accountability and patient safety monitoring, while in Sweden, large-scale public investment in CPD has improved workforce adaptability and retention. These examples indicate that the Polish reform is part of a broader international movement toward regulatory formalization, suggesting that despite initial costs, long-term benefits for both patients and health systems are likely.

In the long term, the formalization of these professions should be assessed along two complementary dimensions. As a legal-regulatory instrument, the Act enhances professional accountability, standardizes qualifications, and improves patient safety. As an economic policy tool, it entails short-term compliance costs but offers potential medium- and long-term benefits in terms of fiscal neutrality, cost-efficiency, and labor market stabilization. This dual perspective confirms that the Act represents not only a milestone in healthcare regulation but also an intervention with measurable economic significance for the Polish health system.

A key limitation of this study is the current lack of publicly available economic data related to the implementation of the 2023 Act. The unavailability of detailed cost and outcome indicators constrains the depth of the economic analysis presented. Future publication of empirical data by governmental or research institutions would significantly expand and refine the economic dimension of this article.

Beyond its national significance, the Act also offers lessons for other EU and OECD countries. It demonstrates the value of aligning new professional regulations with EU directives, thereby facilitating cross-border mobility of healthcare workers. For countries facing similar workforce shortages, the Polish example underlines the importance of integrating CPD and transparent registration systems as tools for both domestic quality improvement and international workforce portability.

Future research should quantify the actual costs and savings resulting from implementation of the Act, including ICT maintenance, training expenses, and quality-of-care outcomes. Comparative longitudinal studies across EU member states could further clarify the relationship between regulatory formalization and healthcare efficiency.
